# Laparoscopic versus open distal pancreatectomy: a single-institution comparative study

**DOI:** 10.1186/1477-7819-12-327

**Published:** 2014-11-05

**Authors:** Yue Zhang, Xue-Min Chen, Dong-Lin Sun

**Affiliations:** Department of Hepatobiliary Surgery, The Third Affiliated Hospital of Soochow University, Changzhou, 213003 China

**Keywords:** Distal pancreatectomy, Laparoscopic distal pancreatectomy, Open surgery

## Abstract

**Background:**

This study was designed to compare clinical outcomes for laparoscopic distal pancreatectomy (LDP) and open distal pancreatectomy (ODP) performed at a single institution.

**Methods:**

This retrospective study included 43 patients who underwent distal pancreatectomy between 2009 and 2013. The patients were divided into two groups based on the surgical approach: the laparoscopic surgery group (n = 20) and the open surgery group (n = 23). All clinical data were analyzed retrospectively.

**Results:**

There were no significant differences in operation time, rate of intraoperative transfusions, complications, or mortality between the two groups. The intraoperative blood loss (210 ± 84.4 mL vs. 420 ± 91.1 mL), first flatus time (1.5 ± 1 d vs. 4 ± 2.5 d), diet start time (2 ± 0.7 d vs. 6 ± 1.8 d), and postoperative hospital stay (8 ± 3.5 d vs. 14 ± 5.5 d) were significantly less in the LDP group than in the ODP group. All patients had negative surgical margins at final pathology. There were no significant differences in the number of lymph nodes harvested (10 ± 2.1 vs. 11 ± 3.2) between the two groups.

**Conclusions:**

LDP is a feasible and safe surgical approach as well as ODP, but has the advantages of an earlier return to normal bowel movements, normal diet, and shorter hospital stays than ODP.

## Background

Minimally invasive surgery has proved to be safe and effective and has largely replaced open surgery in a wide range of procedures. Despite this trend, laparoscopic pancreatic surgery has been slow to gain acceptance. In 1996, new prospects in pancreatic surgery were opened by Gagner [1], who reported his initial experience on five cases of “spleen-preserving” laparoscopic distal pancreatectomy (LDP) for insulinoma. Furthermore, the most frequent of resectable distal pancreatic tumours are currently cystic and endocrine neoplasms, which are often benign and usually diagnosed incidentally during ultrasound examinations carried out in young women. Therefore, the laparoscopic technique is becoming increasingly popular among surgeons to perform distal pancreatectomy. A comparison between open surgery and laparoscopic distal pancreatectomy confirms advantages commonly ascribed to minimal-access surgery such as reduced postoperative pain, faster recovery, and fewer wound-related and general morbidity [2–5]. Although the laparoscopic approach to distal pancreatectomy has become a feasible option over the last few years, it still faces two problems: firstly, sparing the spleen with or without ligation of the splenic vessels, and secondly, controlling the leak from the pancreatic remnant and pancreatic fistula [6]. However, some controversy about its indications and safety concerning long-term oncologic outcome, still exist. The authors of the largest multicentre laparoscopic distal pancreatectomy series commented on their high rate of pancreatic complications when compared with open procedures, especially pancreatic fistula [7].

The aim of this study was to undertake a comparison between LDP and open distal pancreatectomy (ODP). Clinical outcomes were analysed to assess any differences between the LDP and ODP groups.

## Methods

We undertook a retrospective cohort study of the DP in our institution between January 2009 and August 2013 (n = 43). Twenty three (53.5%) patients of them underwent an open technique, while 20 (46.5%) patients underwent a laparoscopic approach. The surgeons could decide whether to perform laparoscopic or open surgery with the informed consent of the patients. Distal pancreas resection was defined as any resection of the pancreas parenchyma starting at the neck or distal to the neck with or without splenectomy and included subtotal resections up to the level of the gastroduodenal artery and superior mesenteric vein. Data collection included patient characteristics, operative details, morbidity, and mortality, postoperative hospital stay, and pathology of specimen.

Postoperative complications were collected and scored according to the Clavien-Dindo complication scale [8]. Complications of Grades I and II were considered minor; those of Grades III to V were considered major. Pancreatic fistula, delayed gastric emptying, and post-pancreatectomy haemorrhage were defined according to the International Study Group of Pancreatic Surgery definitions [9–11]. Oncologic outcomes were analysed for all patients including tumour size (maximum dimension, cm), total number of lymph nodes, number of positive lymph nodes, and margin status.

### Operative technique used for distal pancreatectomy

LDP was performed with the patient in supine and 30° anti-Trendelenburg position with the surgeon standing between the patient’s legs. Basically, five trocars (three 5 mm and two 10 mm trocars) were inserted in the upper abdominal quadrant. We used a supraumbilical cutdown in patients to establish pneumoperitoneum, with a 5 mm port and a 10-mm port in the left upper and left flank quadrants, and two 5 mm ports in the right upper and right flank quadrants. We found that such port placements were ergonomically satisfactory and allowed adequate exposure. Under pneumoperitoneum, the gastrocolic ligament was divided for entrance to the lesser sac using a harmonic scalpel (Harmonic Ace; Ethicon Endo-Surgery, Cincinnati, OH, USA). The mobilization of the pancreas began at the superior border until the proximal splenic artery was visualized. The pancreas was mobilized at the inferior border to visualize the superior mesenteric and splenic veins. After creating a tunnel behind the neck of the pancreas, the pancreas was transected with an endoscopic linear stapler (Endocutter 60 stapler, white or blue cartridge; Ethicon Endo-Surgery). For spleen-preserving procedures, the distal pancreas was freely dissected from the splenic vessels by ligation of the small branches connected to the pancreas using a small Hem-o-lok (Tyco, USA) or a harmonic scalpel. In the case of distal pancreatectomy with splenectomy, the splenic artery and splenic vein were divided. The spleen was resected with the pancreas.

ODP was performed through an upper midline incision. Parenchymal transection was accomplished with staplers and individual pancreatic duct ligation in most patients.

### Follow-up

After surgery, all patients underwent regular follow-up consultations at 3 months, 6 months, and annually thereafter. The follow-ups included clinical examination and CT scan of the thorax and abdomen.

### Statistical analysis

All results are expressed as median and range values. Continuous variables were analysed using Mann-Whitney U-test, whereas categorical variables were analysed using the χ^2^ and/or Fisher’s exact test. A *P* value of <0.05 was considered significant. All statistical analyses were performed using SPSS software for Windows (version 18; SPSS, Inc., Chicago, IL, USA).

## Results

### Baseline patient clinical characteristics

In the study time period, a total of 43 patients underwent distal pancreatectomy at the study institution. Open and laparoscopic distal pancreatectomies were performed in 23 (53.5%) and 20 (46.5%) patients, respectively. Table 1 shows patient information, including age, sex, BMI, tumour size, spleen preservation rate, and American Society of Anesthesiologists score for the two groups. No significant differences in any of these parameters were found between the groups.

**Table 1:** **Clinical demographics of patients in LDP group and ODP group**

Preoperative clinical variables	LDP (n = 20)	ODP (n = 23)
Age (years)	43 ± 11.6	47 ± 13.5
Sex (F/M)	12/8	14/9
ASA	1.5 ± 1	1.5 ± 1
Tumour size (cm)	5.4 ± 2.3	6.8 ± 3.5
BMI (kg/m^2^)	23.8 ± 2.5	22.7 ± 3.3
Spleen preservation		
Yes	10	12
No	10	11

ASA, American Society of Anesthesiologists; ODP, Open distal pancreatectomy; LDP, Laparoscopic distal pancreatectomy; F, Female; M, Male.

### Perioperative outcomes

Comparative analysis of patient’s demographics and intraoperative results are summarized in Table 2. The median operating time in the LDP group was 182 ± 66 min compared with 170 ± 70 min in the ODP group. There were no significant differences between the two groups. No intraoperative blood transfusions were needed. LDP produced a significantly lower amount of intraoperative blood loss (210 mL vs. 420 mL, *P* <0.05), shorter first flatus time (1.5 d vs. 4 d, *P* <0.05), and shorter diet start time (2 d vs. 6 d, *P* <0.05). The total hospital stay was significantly shorter in the LDP group with a median of 8 ± 3.5 days versus 14 ± 5.5 days in the ODP group (*P* <0.05).

**Table 2:** **Operative variables and complications**

	LDP (n = 20)	ODP (n = 23)
Mean operative time (min)	182 ± 66	170 ± 70
Blood loss (mL)	210 ± 84.4	420 ± 91.1*
First flatus time (days)	1.5 ± 1	4 ± 2.5*
Diet start time (days)	2 ± 0.7	6 ± 1.8*
Postoperative hospital stay (days)	8 ± 3.5	14 ± 5.5*
Postoperative complications (%)	25	26
Intra-abdominal abscess (%)	0	0
Delayed gastric emptying (%)	0	0
Pulmonary complications (%)	0	0
Pancreatic fistula	25	26
Grade A	4	4
Grade B	1	2
Mortality	0	0

*P <0.05 compared to LDP group.

ODP, Open distal pancreatectomy; LDP, Laparoscopic distal pancreatectomy.

Postoperative complications occurred in five (25%) versus six (26%) patients in the LDP and ODP groups, respectively. There were no significant differences in postoperative complication rates. A pancreatic leak developed in five (25%) [Grade A = 4, Grade B = 1] patients in the LDP group versus six (26%) [Grade A = 4, Grade B = 2] patients in the ODP group. None of the patients in the LPD group were converted to an open procedure. No perioperative mortality was recorded.

### Oncologic outcomes

Tables 3 and 4 show the pathological characteristics of the two groups. Tumour size did not vary significantly between the LDP and ODP. All patients had negative surgical margins at final pathology. There was no significant difference in the number of harvested lymph nodes (10 vs. 11) between the two groups. In two patients, the pathological findings were consistent with malignant features of a solid pseudopapillary tumour. The malignant features showed local invasion of peripancreatic tissue. There were no significant differences in the pathological characteristics between the two groups.

**Table 3:** **Pathologic diagnosis**

	LDP (n = 20)	ODP (n = 23)
Ductal adenocarcinoma	4	7
Mucinous cystadenoma	3	4
Serous cystadenoma	4	3
Solid pseudopapillary tumour	7	4
Intraductal papillary mucinous		
Neoplasm	1	5
Pancreatic pseudocyst	1	
Total	20	23

ODP, Open distal pancreatectomy; LDP, Laparoscopic distal pancreatectomy.

**Table 4:** **Oncological outcome following LDP versus ODP**

	LDP (n = 20)	ODP (n = 23)
Harvested lymph nodes	10 ± 2.1	11 ± 3.2
Invasion of peripancreatic		
Tissue	2	0
Perineural invasion	0	0
Positive lymph nodes	0	0
Invasion of adjacent organs	0	0
Negative resection margin	20	23

ODP, Open distal pancreatectomy; LDP, Laparoscopic distal pancreatectomy.

The median follow-up was 24 (6–32) months for the LDP group and 30 (12–50) months for the ODP group. Of the malignant cases, recurrence in the liver has occurred in 1 of the LDP patients (20 months after surgery) and in 2 of the ODP patients (13 months and 18 months after surgery, respectively).

## Discussion

The traditional surgical approach to the distal pancreas requires large abdominal incisions and entails possible postoperative complications such as wound infections and incisional hernia. Laparoscopic surgery has the advantage of requiring smaller incisions and less bowel manipulation than does open surgery, thereby reducing pain and analgesic requirements, and facilitating the earlier recovery of bowel function and ambulation [12, 13]. In a review of the English literature, we were unable to find any randomized controlled trials comparing the open and laparoscopic approaches to distal pancreatic tumours. However, multiple retrospective series [14–16] confirm the advantage of LDP over ODP in reporting reduced postoperative pain, shorter hospital stay, more rapid return to normal activity, and better cosmetic results. Laparoscopic pancreatic surgery has been slow to gain popularity, primarily because of technical difficulties. Abu Hilal et al. [17] went further, suggesting that LDP should only be performed in specialized centres and by surgeons with extensive experience in pancreatic and laparoscopic surgery.

In this study, we demonstrated that both LDP and ODP have similar clinical outcomes in terms of operative times, and perioperative complications, but that LDP is associated with advantages such as a shorter hospital stay, lower amount of intraoperative blood loss, and earlier resumption of diet. These results confirm that LDP is safe and effective. Although our study was a retrospective analysis, it involved a single operator at a single centre, thus increasing its reliability.

The most serious complication after a distal pancreatectomy is pancreatic leakage, and the rate of pancreatic leakage after LPD is reported to range from 10% to 33% [18, 19]. In the current study, it occurred in five laparoscopic patients (25%) and in six open patients (26%). This difference was not statistically significant. All the patients with a pancreatic fistula recovered with conservative management. The factors associated with the development of pancreatic leakage were not evaluated in this study. According to a previous article [20], the urgency of the operation and closure of the pancreatic stump were significant factors associated with leakage in OPD. Further studies are necessary to evaluate the factors associated with the development of pancreatic leakage in LDP.

Furthermore, quality of life should be considered when choosing the surgical procedure. Spleen-preserving LDP is thought to be an ideal procedure for a benign tumour. The splenic vessels could be spared in most of our benign cases, which is an obvious advantage for splenic vascularization. As compared with spleen-preserving-LDP, LDP with splenectomy tends to impair quality of life, with frequent higher-grade complications and prolonged hospital stays [21]. Although spleen preservation is feasible even when the splenic vessels are killed, their preservation reduces the risk of abscesses or splenic infarction, which was experienced in 1 patient. We prefer to have the patient in the supine position during the intervention, which allows a routine anatomic approach to the retrogastric cavity. The right lateral position and the initial mobilization of the spleen do not facilitate the surgical manoeuvres of splenic vessel isolation and dissection.

Recently, we have resected four ductal adenocarcinoma with an excellent postoperative course. Despite remaining controversial, laparoscopic resection of ductal carcinomas in our experience was safe and oncologically correct with disease-free resection margins. Nevertheless, the number of cases is still too small to draw definitive conclusions for this particular histotype and laparoscopic resection.

## Conclusions

In conclusion, LDP is feasible and safe. It entails operative times and complication rates similar to those for ODP. In addition, LDP is associated with an earlier return to normal bowel movement and diet and shorter hospital stays than ODP. We believe that this study could provide useful evidence in clinical practice.

## Consent

Written informed consent was obtained from the patient for the publication of this report and any accompanying images.

## Abbreviations

ODP: Open distal pancreatectomy
LDP: Laparoscopic distal pancreatectomy.
